# Bioavailable Soil Phosphorus Decreases with Increasing Elevation in a Subarctic Tundra Landscape

**DOI:** 10.1371/journal.pone.0092942

**Published:** 2014-03-27

**Authors:** Andrea G. Vincent, Maja K. Sundqvist, David A. Wardle, Reiner Giesler

**Affiliations:** 1 Department of Ecology and Environmental Sciences, Umeå University, Umeå, Sweden; 2 Department of Forest Ecology and Management, Swedish University of Agricultural Sciences, Umeå, Sweden; 3 Climate Impacts Research Centre, Department of Ecology and Environmental Sciences, Umeå University, Abisko, Sweden; University of Maryland, United States of America

## Abstract

Phosphorus (P) is an important macronutrient in arctic and subarctic tundra and its bioavailability is regulated by the mineralization of organic P. Temperature is likely to be an important control on P bioavailability, although effects may differ across contrasting plant communities with different soil properties. We used an elevational gradient in northern Sweden that included both heath and meadow vegetation types at all elevations to study the effects of temperature, soil P sorption capacity and oxalate-extractable aluminium (Al_ox_) and iron (Fe_ox_) on the concentration of different soil P fractions. We hypothesized that the concentration of labile P fractions would decrease with increasing elevation (and thus declining temperature), but would be lower in meadow than in heath, given that N to P ratios in meadow foliage are higher. As expected, labile P in the form of Resin-P declined sharply with elevation for both vegetation types. Meadow soils did not have lower concentrations of Resin-P than heath soils, but they did have 2–fold and 1.5–fold higher concentrations of NaOH-extractable organic P and Residual P, respectively. Further, meadow soils had 3-fold higher concentrations of Al_ox_ + Fe_ox_ and a 20% higher P sorption index than did heath soils. Additionally, Resin-P expressed as a proportion of total soil P for the meadow was on average half that in the heath. Declining Resin-P concentrations with elevation were best explained by an associated 2.5–3.0°C decline in temperature. In contrast, the lower P availability in meadow relative to heath soils may be associated with impaired organic P mineralization, as indicated by a higher accumulation of organic P and P sorption capacity. Our results indicate that predicted temperature increases in the arctic over the next century may influence P availability and biogeochemistry, with consequences for key ecosystem processes limited by P, such as primary productivity.

## Introduction

Phosphorus (P) is an important macronutrient in subarctic tundra, where it is often co-limiting with nitrogen (N), and is sometimes the main limiting nutrient for plant growth [1]–[3]. The main source of plant-available, inorganic P (hereafter ‘available P’) in subarctic tundra is the biological mineralization of organic P [1], [4], [5]. Most soil P in the surface of tundra soils is organic [1], [4], [6] and is dominated by highly labile compounds [7]. Temperature is one of the main controls of organic matter decomposition in the arctic [8]–[10], meaning it is likely to constrain organic P mineralization and the supply of available P for plants. As such, warming experiments often show increases in P mineralization and/or plant P availability [9], [11], [12]. Likewise, an increase in foliar and litter P concentrations, together with a decrease in foliar and litter N to P ratios (indicative of greater relative P availability), have been observed with decreasing elevation (thus increasing temperature) in subarctic tundra [13]. The predicted annual average air temperature increases of 3–5°C in the subarctic during this century [14]–[16] could therefore influence the availability of P. Current knowledge on the distribution of different P forms in tundra landscapes and how they may be affected by temperature is limited, despite this information being crucial for understanding future temperature effects on bioavailable P.

Elevational gradients are powerful tools for studying how temperature and associated climatic factors, which shift with elevation, influence ecosystems properties and processes [17]–[21]. As such, an increasing number of studies in a wide range of ecosystems have used elevational gradients to study how temperature affects ecological processes [22]–[24] including in the subarctic [13], [25]–[28]. Elevational gradients also provide excellent opportunities for exploring the impacts of temperature on the availability of soil P over larger spatial scales and timeframes than what is possible through conventional experiments [19]. There are, to our knowledge, no studies available on the responses of different P pools to elevation in subarctic or arctic tundra. These landscapes are biogeochemically heterogeneous as a consequence of spatial variation in topography and plant community structure [29], [30]. This results in high spatial variation in P availability [6], [25] and in the concentrations of Al and Fe [6], [31], which influence P availability [32], [33]. The Fennoscandian tundra therefore consists of a mosaic of highly contrasting vegetation types. Specifically, heath vegetation occurs on soils with low pH and N availability and is dominated by slow-growing dwarf-shrubs, while meadow vegetation grows on soils that are more N-rich and is dominated by faster-growing herbaceous species [25], [29], [30]. The biogeochemistry of P differs between the two vegetation types [6] and soil phosphate concentrations and plant foliar N to P ratios suggest that the relative importance of P versus N limitation is greater in meadow than in heath [6], [13], [25]. As such, obtaining a representative picture of how P biogeochemistry varies in the subarctic requires explicit recognition of both vegetation types.

In this study, we used a well-established elevational gradient [13], [25], [26], [34] in which both heath and meadow vegetation types occur at all elevations, to study the effects of elevation-associated variation in temperature on P availability and biogeochemistry in a subarctic ecosystem. We also used this gradient to examine whether previously reported changes in foliar and litter P contents and N to P ratios with elevation are matched by shifts in the concentration of P fractions of different lability. To determine P fractions we used the Hedley fractionation method [35]–[38], an approach widely used to determine landscape-level variation in P availability and dynamics [6], [39], [40]–[42]. Additionally, we examined if other known drivers of P availability, such as soil P sorption capacity and Al and Fe concentration, influence the distribution of P fractions across the gradient for both the heath and meadow vegetation. Specifically, we tested the hypotheses that (1) The concentrations of labile P fractions decline with elevation (and therefore temperature) regardless of vegetation type, (2) Across all elevations, meadow soils have consistently lower concentrations of labile P than heath soils, together with higher concentrations of Al and Fe and higher soil P sorption capacity. By addressing these hypotheses we aim to better understand how temperature changes, such as those that are expected through climate warming, may affect P availability across two dominant vegetation types in subarctic tundra ecosystems.

## Materials and Methods

### Ethics statement

**Figure 1:**
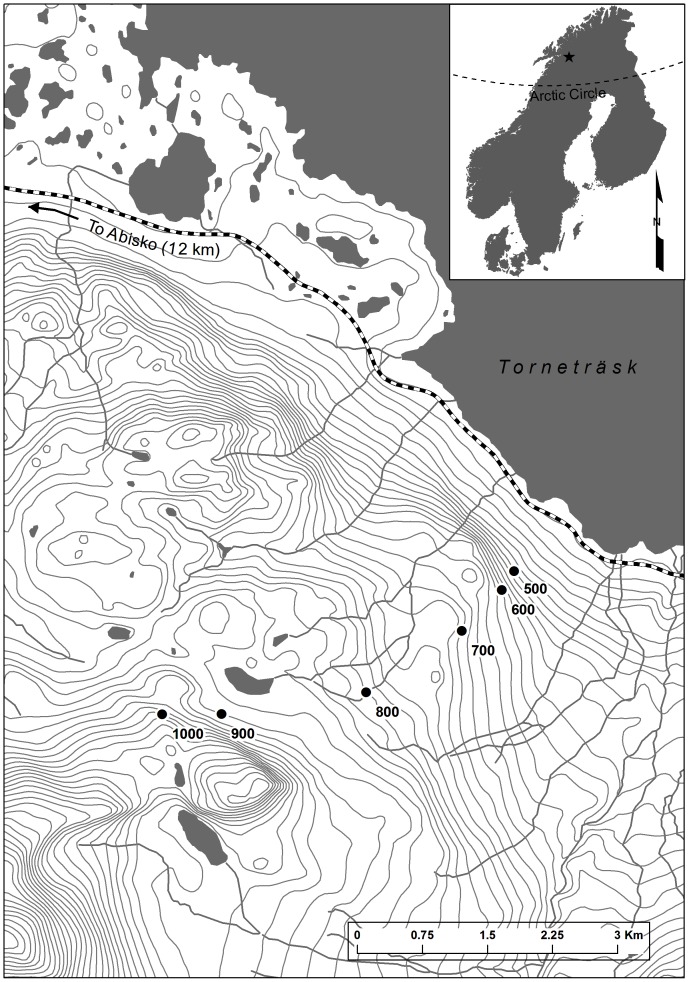
Location of the study elevational gradient. Filled black circles indicate each of the six study sites, ranging in elevation from 500 to 1000

This study was carried out across an elevational gradient ranging from 500 to 1000 m above sea level (a.s.l.) along the north-east facing slope of Mount Suorooaivi (1193 m), located approximately 20 km south-east of Abisko, northern Sweden (68°21′ N, 18°49′ E), as described in [25]. Figure 1 shows a map with the location of the study site and the elevational gradient. No part of this gradient is located within national reserves and the land is public and not government protected. We confirm that all national and international rules were observed during the field work. This investigation did not involve measurements on animals or humans. The soils collected for this research were sampled at very small spatial scales and thus had negligible effects on ecosystem functioning. We have no commercial interests or conflicts of interest in performing this work.

### Study site

The mean annual precipitation in the area, measured at the Abisko Scientific Research Station, was 310 mm for the period 1913–2000, with the highest mean monthly precipitation in July (51 mm) and the lowest in April (12 mm) [43]. The treeline in this area is formed by *Betula pubescens* spp. *czerepanovii* (mountain birch) and is located at 500–650 m a.s.l. at the study site. Two types of vegetation, heath and meadow, grow in a mosaic across the study area and co-occur on all elevations, with the meadow generally found in shallow depressions. The heath is dominated by ericaceous dwarf-shrubs such as *Vaccinium vitis-idaea*, *V. uliginosum* spp. *uliginosum*, *Empetrum hermaphroditum* and *Betula nana*. The meadow vegetation is dominated by graminoids such as *Deschampsia flexuosa* and *Anthoxanthum alpinum*, herbs such as *Saussurea alpina*, *Viola biflora* and *Solidago virgaurea*, and sedges, notably *Carex bigelowii* [25]. The bedrock is comprised of salic igneous rocks and quartic and phyllitic hard schists. The soils are podzols at lower elevations and cryosols at higher elevations. For more details on the study system see Table S1 and [13], [25].

In the summer of 2007, four replicate plots (2×2 m) were established in each of the two vegetation types in each of six elevations (every 100 m ranging from 500 to 1000 m) rendering a total of 48 plots as described by [13], [25]. To minimize pseudoreplication within each elevation, the average distance between each plot and the next nearest plot was c. 15 m (with the mean distance between the two most distant plots in each vegetation type being c. 100 m). Because the microtopography, hydrology, and soil fertility of these communities is highly spatially heterogeneous over short distances (i.e. in the order of a few metres) [29], it is expected that the 15 m distance among plots is enough to ensure adequate independence among them [13], [25]. Plots at the 500 m elevation site were located in open birch forest, plots at 600 m were situated immediately above the forest line, and plots from 700 m to 1000 m were devoid of trees [25]. Monthly mean air temperatures during August 2009 at 500 m, 700 m and 1000 m at the study site were 12.1°C, 11.8°C, and 9.9°C, respectively. The daily mean temperature across the elevational gradient during the growing season of 2009 is given in Figure S1; similar data for the previous year (2008) is also given in Figure S2 [25]. Elevational gradients of this type serve as useful natural experiments to inform on the effects of temperature on ecological properties when other potentially co-varying factors can be kept constant [17], [18]; as such, all plots in our study have the same aspect (north-east facing slope), parent material, and slopes of 4–18°.

### Soil sampling

Humus soils were sampled on August 4, 2009, in a 1×1 m subplot inside each 2×2 m plot. For each plot, a minimum of four 4.5 cm diameter cores were sampled to the full humus depth to ensure a total sample volume of approximately 0.3 L humus, and humus depth was recorded. The humus depth (mean ± standard error) across all heath plots was 5.4±0.2 cm (for each elevation mean depths are 6.3, 6.1, 5.0, 4.0, 5.8, and 5.4 cm starting at 500 m.a.s.l. in ascending order), and for meadow plots it was 3.0±0.6 cm (starting at the 500 m.a.s.l. site and in ascending order, 7.2, 2.2, 1.7, 1.1, 3.2, and 2.8 cm). Within each plot, the cores were sieved (2 mm mesh) in the field to homogenize the samples, and combined to yield a single bulked sample per plot. Samples were sealed in polyethylene bags and transported to the laboratory on the same day as sampling. From each sample, a subsample was immediately stored at 2°C (<48 h) and the remaining portion was frozen at −20°C.

### Hedley fractionation

**Figure 2:**
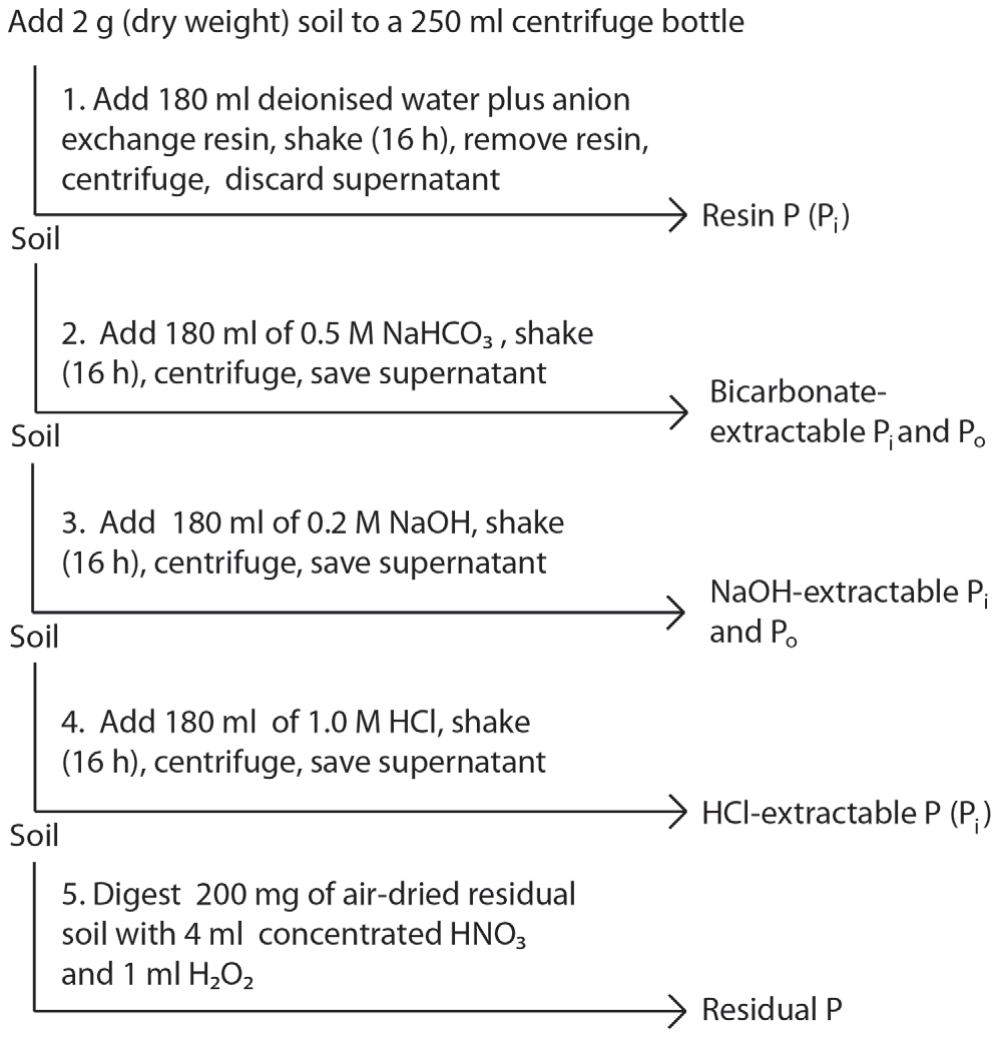
Flow chart showing the five steps involved in the sequential phosphorus fractionation used in this study.

In order to characterize soil P composition, we performed a five-step sequential extraction [38] with some modifications [44], [45], outlined in Figure 2. This method was chosen because it provides a direct estimate of the lability of different operationally-defined P pools, and is the most commonly used method to investigate differences in P availability and dynamics in natural soils [35]–[37], [46]. In Step 1, 2 g (dry weight) of humus soil were combined with 180 mL deionized water and one 9×62 mm anion exchange membrane (hereafter called ‘resin’) (55164 2S, BDH Laboratory Supplies, Poole, England) [47] in a 250 mL centrifuge bottle and shaken overnight (16 h, 150 rpm). The following day, the resins were removed and the sample centrifuged (14 000 *g*, 15 min, 10°C), after which the supernatant was discarded and the remaining soil used in Step 2. The resin was transferred to a bottle and eluated on a shaker (1 h, 150 rpm) with 40 mL NaCl. The eluate was immediately stored at −20°C until further analysis of this resin-extractable P fraction, hereafter referred to as ‘Resin-P’. In Step 2, the soil remaining from Step 1 was combined with 180 mL 0.5 M NaHCO_3_, set on a shaker (16 h, 150 rpm), centrifuged (14 000 *g*, 15 min, 10°C), and 40 mL of the supernatant removed and stored at −20°C for determination of organic and inorganic NaHCO_3_-extractable P (hereafter referred to as ‘Bic-P_o_’ and ‘Bic-P_i_’, respectively). In Step 3, the soil remaining from Step 2 was extracted with 0.2 M NaOH following the same procedure as in Step 2 and stored at −20°C for further analysis of organic and inorganic NaOH-extractable P (hereafter referred to as ‘NaOH-P_o_’ and ‘NaOH-P_i_’, respectively). In Step 4, the soil remaining from Step 3 was combined with 1.0 M HCl, following the exact same extraction procedure as in Steps 2 and 3 and the supernatant stored at −20°C for further analysis of HCl-extractable P (hereafter, ‘HCl-P’). In the final and fifth step, the soil remaining from Step 4 was washed with 180 mL of deionised water by shaking for 1 h, centrifuging, and discarding the supernatant, after which the soil was set to air dry at room temperature. This residual air-dried soil was ground in a ball mill and a 200 mg subsample was combined with 4 mL of concentrated nitric acid (HNO_3_) and 1 mL of hydrogen peroxide (H_2_O_2_) (soil to solution ratio 1∶40) and digested in a microwave (Mars XPress, CEM, Germany). This constitutes the ‘Residual-P’ fraction.

Total labile P consists of resin-extractable and bicarbonate-extractable P [48]. Resin-P is well correlated with P uptake by plants [49], [50], has a rapid turnover and high bioavailability, and consists of P in a form that can exchange freely between the solid phase of the soil and the soil solution [35], [51]. Bicarbonate-P_i_ (Bic-P_i_) has similar sources to resin-P, turns over fast and is also bioavailable in the short-term [35], while bicarbonate-P_o_ (Bic-P_o_) is easily mineralizable and supplies plant-available P. Hydroxide-extractable P_i_ (NaOH-P_i_) and P_o_ (NaOH-P_o_) are associated with Al and Fe phosphates, have lower plant availability, and longer turnover times. HCl-extractable P represents calcium-bound P_i_ and is often taken to represent P associated with primary minerals; it is also considered to be more stable [48]. By using extractants of different strength, this method is considered to quantify pools according to their ‘lability’ to plants [48].

### Sorption index

In order to estimate the relative P sorption capacity of each soil sample from each plot, we used a single point P sorption method [52]. For each sample, 2 g (dry weight) of soil were weighed into each of two 60 mL bottles respectively, and 40 mL of 100 mM KCl was added to each bottle. For one of the two bottles, 1.6 mg P g soil^−1^ (dry weight basis, equivalent to 50 mmol kg^−1^ soil) of phosphate (KH_2_PO_4_) was added, which yielded two suspensions per soil sample: one with added P (spiked) and one without added P (unspiked). The suspensions were shaken for 24 h, filtered (Munktell 00H filter paper, pore size approx. 1 μm; Grycksbo, Sweden) and the amount of phosphate in each of the two filtrates was determined colorimetrically. The amount of sorbed P was estimated as the difference between the phosphate concentration in spiked and unspiked samples. We calculated the P sorption capacity using the equation
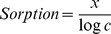
where *x* is the quantity of P sorbed onto soil constituents and *c* is the equilibrium P concentration in the soil +P solution [52]. Thus, a high index indicates a high P sorption capacity.

### Determination of P in extracts

All Hedley fractionation and sorption index extracts were analysed for molybdate-reactive P using a flow injection analyzer (FIA) (FIAstarTM 5000 Analyzer, FOSS Analytical AB, Höganäs, Sweden). The NaHCO_3_ and NaOH solutions were diluted by a factor of 5–10 and amended with sulphuric acid (20 μl concentrated H_2_SO_4_ to 5 mL diluted extract) in order to precipitate organic material, after which they were centrifuged and the supernatant analyzed by FIA. Since the NaHCO_3_ and NaOH extracts were colored, the measured P concentration was corrected by subtracting the effect of the color in the analysis. Total P in the NaHCO_3_ and NaOH extracts was determined after digestion with acidified potassium persulfate (K_2_S_2_O_8_), and inorganic P was analysed as above. The concentration of organic P was calculated as the difference between total and inorganic P. Phosphorus in the digests was analysed as above. Total soil P was calculated as the sum of all P fractions measured in the Hedley fractionation [6], [39], [44], [53] and we refer to it hereafter as ‘Total P’. Since all of our samples are from organic soils, we express the concentrations of all P fractions as mg kg^−1^ soil dry weight, which would be equivalent to expressing them on a per unit organic matter basis.

### Aluminium and iron

Aluminium (Al) and iron (Fe) concentrations in the soil samples were determined following extraction by 0.2 M acid oxalate (C_2_H_8_N_2_O_4_) adjusted to a pH of 3 [54]. A subsample of 0.5 g dry weight of soil from each sample was combined with acid oxalate solution in a 1∶30 soil to solution ratio and shaken on an orbital shaker for 4 h in the dark. Extracts were subsequently filtered (00H, Munktell Filter AB, Grycksbo, Sweden) and stored at 5°C (∼2 months) following analysis of oxalate extractable Al and Fe (hereafter referred to as Al_ox_ and Fe_ox_, respectively) determined by inductively-coupled plasma optical-emission spectroscopy (ICP-OES) (Perkin Elmer). The oxalate extractant is assumed to release exchangeable Al and Fe and dissolve non-crystalline and poorly crystalline oxides of Al and Fe (i.e. organic and amorphous Al and Fe forms), which are major P sorbents in non-calcareous soils [55]. Concentrations of Al_ox_, Fe_ox_, and all P fractions are expressed as mg kg^−1^ soil dry weight on the basis of oven-dried soils (105°C, 24 h).

### Statistical analysis

We used multivariate ANOVA (MANOVA) for the entire dataset given the large number of response variables measured per soil sample. We followed a significant MANOVA with a two-way ANOVA to test for the effect of elevation and vegetation type and their interaction on each response variable [13], [25], [26]. We chose ANOVA because it is the most powerful way of detecting significant responses to the underlying gradient even when these responses are not unidirectional or simple [56]. To further explore the effects of elevation within vegetation types, one-way ANOVA testing for the effect of elevation was performed separately for both heath and meadow. Where significant effects of elevation were found, data were further analyzed for differences among means using Tukey's honestly significant difference (h.s.d.) at *p* = 0.05. Tukey's h.s.d. was chosen because it reduces Type I error when multiple comparisons are being performed [57], [58].

In order to account for potential effects of co-variation of soil P sorption capacity with elevation on labile P concentrations, we divided the concentration of each of the three labile components (i.e. Resin-P, Bic-P_i_, and Bic-P_o_) for each soil sample by the sorption index for that sample. We then performed separate one-way ANOVA testing for the effect of elevation on the transformed data, followed by Tukey's h.s.d. at *p* = 0.05 after a significant ANOVA result. We used Pearson's correlation to test for the relationship between Al_ox_ and Fe_ox_; linear regression was used to test for the relationship between soil P fractions with Al_ox_ + Fe_ox_, sorption index and temperature. When required, data were transformed to conform to the assumptions of parametric tests. For statistical analyses we used SPSS PASW Statistics 18.0 and R version 3.3.0 (www.r-project.org).

## Results

### Effects of elevation

**Figure 3:**
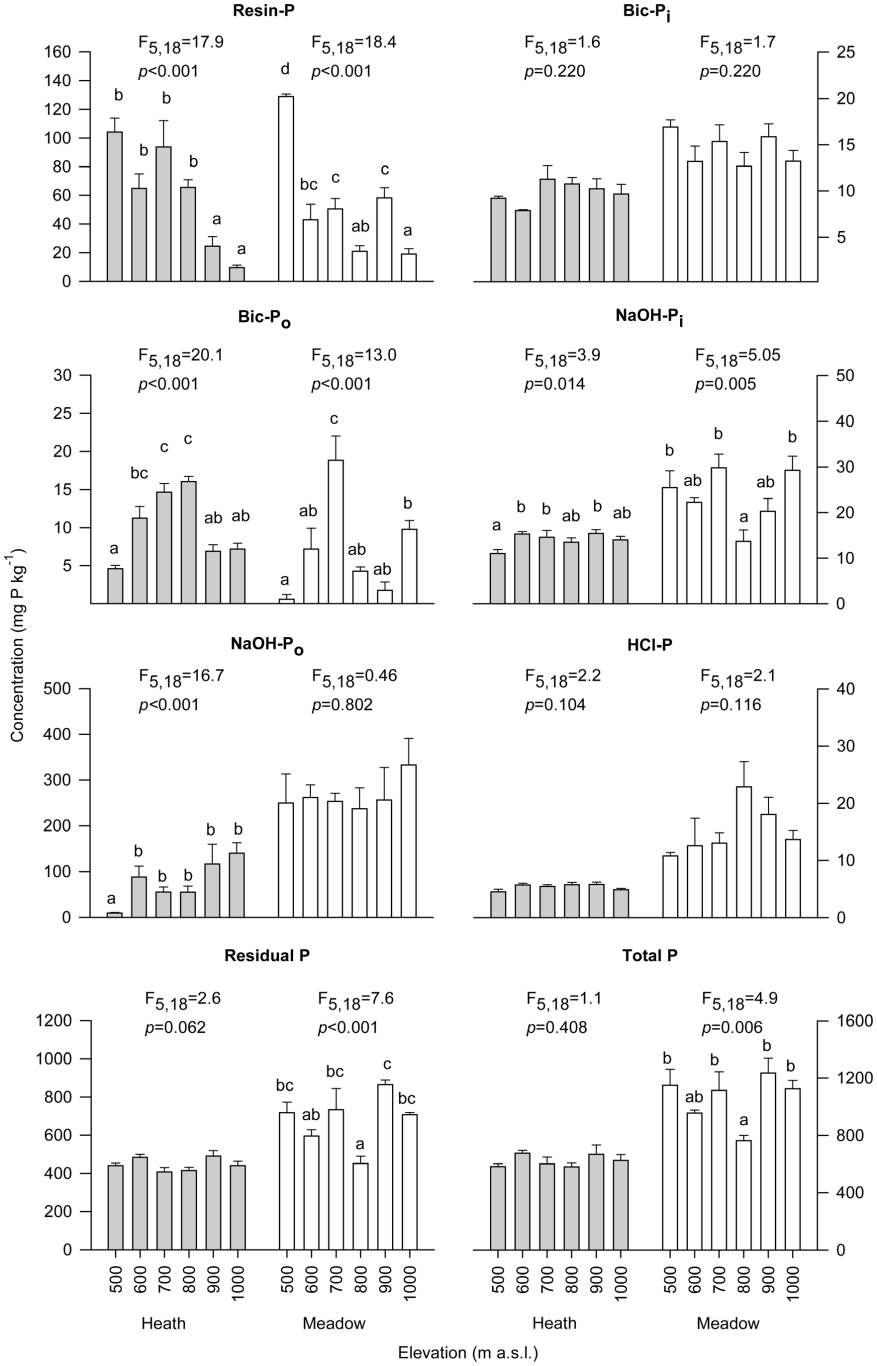
Concentration of phosphorus fractions in humus soils in contrasting vegetation types across an elevational gradient. Soils were collected in subarctic heath and meadow vegetation along an elevational gradient (500–1000 m) in Abisko, Sweden. Panels represent phosphorus fractions extractable with: anion-exchange resins (Resin-P); NaHCO_3_ (inorganic fraction - Bic-P_i_; and organic fraction - Bic-P_o_); NaOH (inorganic fraction - NaOH-P_i_; and organic fraction - NaOH-P_o_); HCl (HCl-P fraction); non-extractable P (Residual P fraction); and Total P (arithmetic sum of all P fraction). Bars represent mean concentration (+1 SE) for four plots; for each P fraction and within each vegetation type, F and *p* values (with d.f.) are from a one-way ANOVA testing for the effect of elevation within each vegetation type, and bars topped with the same letter do not differ at *p* = 0.05 (Tukey's h.s.d.). Note the difference in y-axis scales.

**Figure 4:**
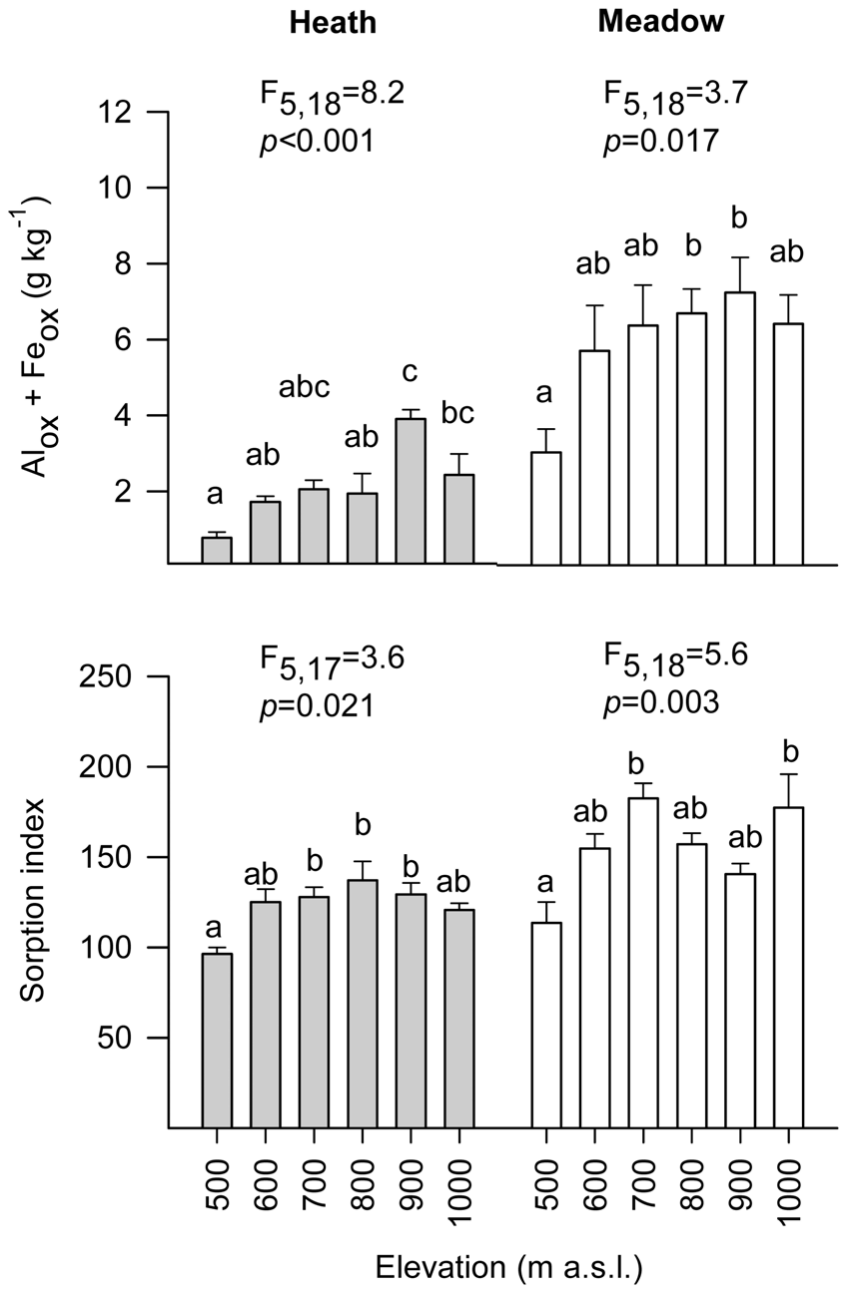
Concentration of aluminum and iron, and phosphorus sorption index for humus soils across an elevational gradient. Soils were collected in subarctic heath and meadow vegetation types along an elevational gradient (500–1000 m) in Abisko, Sweden. The top panel represents the concentration of the sum of oxalate-extractable Al and Fe (Al_ox_ + Fe_ox_), the bottom panel represents the soil phosphorus sorption index. Bars represent mean values (+1 SE) for four plots; within each vegetation type, bars toped with the same letter do not differ at *p* = 0.05 (Tukey's h.s.d.) after a significant ANOVA (ANOVA results in Table 2).

Overall, Residual-P was the most abundant P fraction in humus across all elevations and vegetation types (59−76% of total soil P), followed by NaOH-P_o_ (1.6–31%), and Resin-P (1.5–18%) (Figure 3, Table S2). Total labile P (i.e. the sum of Resin-P, Bic-P_i_ and Bic-P_o_) represented 4–20% of total soil P (Table S2). The concentrations of the remaining fractions ranged from 0.8–3.0% of total soil P (Table S2). The most unidirectional effect of elevation was found for Resin-P, for which the highest concentrations were recorded at the lowest elevation and the lowest concentrations at the highest elevation, for both vegetation types (Figure 3, Table 1). Resin-P concentrations at the highest elevation were 7–fold and 11–fold lower than at the lowest elevation for heath and meadow, respectively. Total labile P trends mirrored those of Resin P, concentrations at the highest elevation (1000 m) in heath were less than one fifth of those recorded at the 500 m (lowest elevation) and 700 m sites. In meadow, Total labile P concentrations at the highest elevation were less than one third of those recorded at the lowest elevation. While elevation also had a significant effect on all other soil properties except Bic-P_i_ and HCl-P (which was almost significant) (Table 1, Table 2), there were no simple unidirectional trends with elevation for any of the other P fractions measured (Figure 3) or for Al_ox_ + Fe_ox_ or the sorption index (Figure 4). After concentrations were divided by the sorption index, the unidirectional elevational trend remained for Resin-P and the non-unidirectional effect remained for Bic-P_o_; further, a significant effect of elevation emerged for Bic-P_i_, through it being highest at the lowest elevation for the meadow (Figure S3). When all elevations and vegetation types were combined, Resin-P was significantly and negatively related with both Al_ox_ + Fe_ox_ and the Sorption Index, although the relationship was weak (Table 3). Significant positive relationships were observed between most other P fractions and Al_ox_ + Fe_ox_ and Sorption Index, the strongest being for NaOH-P_o_ and HCl-P (Table 3). Finally, Resin-P and Total labile P were significantly positively related with temperature in both the heath and meadow vegetation (Table S3), as was the case for pH in the heath. NaOH-P_o_ was negatively correlated with temperature in the heath (Table S3).

**Table 1:** Effect of vegetation type and elevation as determined by multivariate analysis of variance (MANOVA) (F- values, with *p* in parenthesis) and two-way ANOVA on the concentration (mg kg^−1^) of different phosphorus (P) fractions in humus soils along an elevational gradient in Abisko, Sweden.

	ANOVA results
Variables	Vegetation type (V)	Elevation (E)	V x E interaction
Multivariate analyses			
MANOVA	5.602 (<0.001)	23.34 (<0.001)	2.89 (<0.001)
Univariate analysis			
Resin P^a^	0.140 (0.710)	27.4 (<0.001)	8.8 (<0.001)
Bic-extractable P_i_	47.5 (<0.001)	1.8 (0.147)	1.4 (0.255)
Bic-extractable P_o_	12.7 (0.001)	21.5 (<0.001)	7.6 (<0.001)
Total labile P^b^	0.94 (0.339)	27.6 (<0.001)	7.6 (<0.001)
NaOH-extractable P_i_ a	89.3 (<0.001)	4.6 (0.002)	5.1 (0.001)
NaOH-extractable P_o_ a	147.8 (<0.001)	11.5 (<0.001)	8.2 (<0.001)
HCl-extractable P	59.0 (<0.001)	2.4 (0.058)	1.8 (0.142)
Residual P^a^	104.7 (<0.001)	7.9 (<0.001)	4.7 (0.002)
Total P^a^^,^ c	160.5 (<0.001)	4.0 (0.005)	2.7 (0.038)

For all variables, degrees of freedom for V = 1,36, E = 5,36, V*E = 5, 36.

Bic  =  Bicarbonate; P_i_ =  inorganic P; P_o_ =  organic P.

a Data were log transformed prior to analysis.

b Sum of Resin P, Bic-extractable P_i_ and Bic-extractable P_o_.

c Sum of all sequentially extracted P fractions.

**Table 2:** Effects of vegetation type and elevation as determined by ANOVA (F- values, with *p* values in parenthesis) on phosphorus sorption index and the concentration of oxalate-extractable Al and Fe (Al_ox_ + Fe_ox_) in humus soils along an elevational gradient in Abisko, Sweden.

	ANOVA results
Variables	Vegetation type (V)	Elevation (E)	V x E interaction
Al_ox_ + Fe_ox_ (mg kg^−1^)^a^	107.9 (<0.001)	8.8 (<0.001)	0.6 (0.666)
Sorption index	36.3 (<0.001)	7.2 (<0.001)	2.4 (0.060)

Degrees of freedom (d.f.) for V = 1,36, E = 5,36, V*E = 5, 36 for Al_ox_ + Fe_ox_; and d.f. for V = 1,35, E = 5,35, V*E = 5, 35 for Sorption index.

a Data were square-root transformed prior to analysis.

**Table 3:** Linear regressions between the sum of oxalate-extractable aluminium and iron (Al_ox_+ Fe_ox_) and the Sorption Index with phosphorus (P) fractions in humus soils along an elevational gradient in Abisko, Sweden.

P fraction	Al_ox_+Fe_ox_	Sorption Index
	R^2^ (*p*-value)	Direction	R^2^ (*p*-value)	Direction
Resin P	**0.165 (0.002)**	**Negative**	**0.136 (0.006)**	**Negative**
Bic-P_i_	**0.264 (<0.001)**	**Positive**	**0.124 (0.009)**	**Positive**
Bic-Po	0.002 (0.305)	N/A	0.078 (0.032)	**N/A**
Total labile P^a^	**0.138 (0.005)**	**Negative**	**0.070 (0.039)**	**N/A**
NaOH-P_i_	**0.241 (<0.001)**	**Positive**	**0.385 (<0.001)**	**Positive**
NaOH-P_o_	**0.467 (<0.001)**	**Positive**	**0.356 (<0.001)**	**Positive**
HCl P	**0.654 (<0.001)**	**Positive**	**0.223 (<0.001)**	**Positive**
Residual P	**0.352 (<0.001)**	**Positive**	**0.118 (0.010)**	**Positive**
Total P^b^	**0.419 (<0.001)**	**Positive**	**0.218 (<0.001)**	**Positive**

Values are for heath and meadow vegetation data combined. Degrees of freedom for all P pools are 1,46 for Al_ox_+Fe_ox_; and 1,45 for the Sorption Index due to one missing value.

a Sum of Resin P, Bic-extractable P_i_ and Bic-extractable P_o_.

b Sum of all sequentially extracted P fractions.

### Effects of vegetation

**Figure 5:**
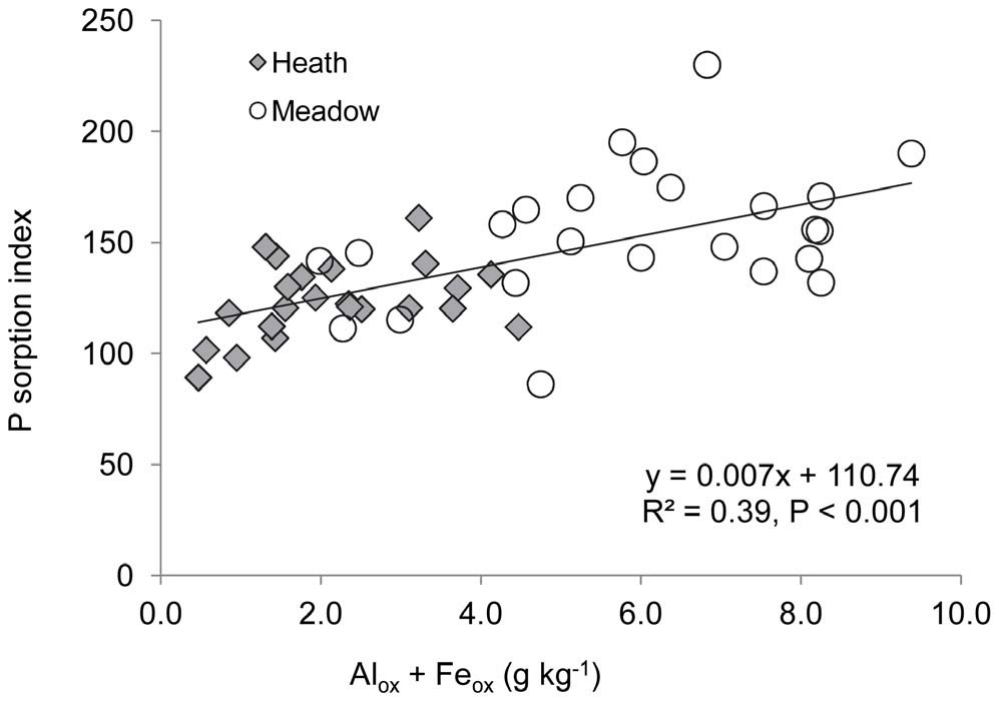
Relationship between phosphorus sorption capacity and metal concentration in humus soils across an elevational gradient. Sorption index versus the sum of oxalate-extractable Al and Fe (Al_ox_ + Fe_ox_) in subarctic heath and meadow vegetation types across an elevational gradient in Abisko, Sweden.

The concentrations of all P fractions except Resin and Total labile P were significantly different between meadow and heath, and of these all were highest in the meadow except for Bic-P_o_ (Table 1, Figure 3). The NaOH-P_o_ fraction had the largest difference in concentration between the two vegetation types (Figure 3); it was on average three–fold higher in meadow than in heath and represented ∼25% of total P in the meadow but only ∼13% in the heath. The concentrations of Al_ox_ and Fe_ox_ were highly correlated with each other (*R* ^2^ = 0.951, *p*<0.001), and their sum (Al_ox_ + Fe_ox_) was on average three times higher in meadow than in heath (Figure 4). The sorption index was also significantly higher in meadow than in heath, on average by 20% (Figure 4, Table 2). Additionally, sorption index was significantly positively related to Al_ox_ + Fe_ox_ (Figure 5). After concentrations were divided by the sorption index, Bic-P_i_ and Bic-P_o_ remained highest in the meadow and heath, respectively, and Resin-P was also highest in the heath (Figure S3). There was an interactive effect between vegetation type and elevation for all P fractions except Bic-P_i_ and HCl-P, meaning that soil P composition responds differently to changes in elevation depending on vegetation type (Table 1, Figure 3).

## Discussion

### Effects of elevation on the concentration of labile P fractions

The concentrations of labile P fractions were hypothesized to decline with increasing elevation and associated declines in temperature, regardless of vegetation type. We consider Resin-P, Bic-P_i_ and Bic-P_o_ to represent the most labile P fractions [35]; Resin-P is well correlated with plant P uptake [49], [59], and is considered to be the most highly bioavailable fraction [35], [51]. Further, Bic-P_i_ is considered to derive from similar sources to Resin-P, while Bic-P_o_ to be easily mineralized [35], [48]. These three fractions are discussed separately given the differences in their concentration and dynamics observed in this study. Partially in line with our predictions, Resin-P concentration declined with elevation in both heath and meadow. However, for this P form there was also a strong interactive effect of vegetation × elevation, meaning that this pattern of decline differed between the two vegetation types and that elevation (and thus temperature) effects on P availability therefore depend on vegetation type. Concentrations of Bic-P_i_ and P_o_ did not show any unidirectional trends with elevation, but given that they occurred in much lower concentrations, the overall trend is still one of declining bioavailable P with increasing elevation. In some organic soils, the concentration of bioavailable inorganic P has been shown to be influenced by Al and/or Fe concentration and soil P sorption capacity [60]–[62]. While Al_ox_ + Fe_ox_ concentrations and the sorption index in our study sites differed across elevations (Figure 4) and were both weakly negatively correlated with Total labile P (Table 3), the elevational trends in Resin-P remained even after correcting for the sorption index (Figure S3), suggesting that they are largely explained by factors other than sorption.

A number of factors could explain the observed decrease in Resin-P with elevation. Organic P (as NaOH-extractable and Residual P) is the dominant form of P in these humus soils and enzymatic hydrolysis of organic P is a likely driver for the release of bioavailable inorganic P, as has been shown for Alaskan tundra [1]. Temperature is the strongest driver of soil enzyme activity in the subarctic [63] and warming experiments show that even a relatively small increase in temperature (1.2–1.7°C) can cause a large increase in organic P mineralization in alpine ecosystems [64]. The average air temperature difference across our elevational gradient during the vegetation growing season is about 2.5–3.0°C (Figures S1 and S2), which would be sufficient to explain the differences in labile P we observed. This suggests that the decline in temperature associated with increasing elevation is an important driver of the elevational decline in Resin-P observed and is consistent with our hypothesis. However, further information on organic P mineralization processes, for example using mineralization and soil enzyme studies together with ^31^P nuclear magnetic resonance (NMR) spectroscopy to characterize soil organic P [65], [66], would be useful to increase our mechanistic understanding of the trends observed here. Our results are in line with the few studies that have measured labile inorganic P concentrations at different elevations in subarctic tundra, as they report lower concentrations of 1.0 M KCl-extractable P at high elevations (1150 and 1000 m) than at low elevations (450 and 500 m) [25], [67]. Our results are also consistent with [13] who showed a decrease in foliar P concentrations and an increase in foliar N to P ratios with elevation independent of vegetation type. Decreasing organic P mineralization with increasing elevation should lead to the accumulation of soil organic P, which was the case for NaOH-P_o_ in heath (Figure 3). However, a greater accumulation of soil organic P with elevation may have been negated to some extent by declining primary productivity, as is observed with increasing elevation both in the vicinity of the study area [28] and globally [19].

Temperature can affect P availability directly via effects on microbial mineralization [68] and soil process rates [69] but also indirectly by influencing factors that affect soil processes such as plant [70] and microbial [71] community composition. Temperature variation across the elevational gradient studied here has previously been shown to be related to a range of variables including soil pH, total N and ammonium concentration, C to N ratio, vegetation density, plant and microbial community composition, and fungal to bacterial ratios [25]. As such, many of the soil and vegetation properties that vary along the elevational gradient are likely to represent indirect temperature controls on P availability. Our interpretation is supported by many other studies that have used elevational gradients to understand how temperature affects ecological properties and processes [17], [22]–[25], [72]–[75].

To our knowledge, this is the first time that a Hedley P fractionation analysis has been carried out along a subarctic elevational gradient [6] and the two most comprehensive reviews on P fractionation lack data for these ecosystems [35], [36]. These data are necessary to constrain soil P pools in terrestrial biogeochemical models, which are invaluable to understand the processes controlling P cycling and the role of P in driving terrestrial plant productivity [36]. The dominance of organic and residual P fractions (∼87% of total soil P on average) and the concentrations of Resin-P measured in these soils are comparable with previous findings for other high latitude tundra ecosystems [1], [4], [6]. Additionally, the highest Resin-P concentrations measured (i.e. at the lowest elevations) were around two thirds of those reported for a Swedish boreal forest humus with low P sorption capacity [39]. While the concentration of Total labile P (sum of Resin P and Bicarbonate-extractable P) is considered to be low in tundra relative to other ecosystems [1], [4]–[6], our results show that this may not necessarily hold when a wide range of elevations is considered. The 3–5 fold variation in Total labile P concentrations that we found along this elevational gradient encompasses the whole range of Total labile P concentrations recently reported in a world synthesis of Hedley P fractionation studies in natural ecosystems that spans 11 soil orders [36]. Our results further suggest that because small changes in elevation (and thus temperature) were associated with large changes in available P, increases in temperature according to current climate change predictions [14]–[16] may have a significant impact on future P availability in arctic tundra.

### Vegetation differences and distribution of different P fractions

We hypothesized that the concentration of labile P fractions (Resin-P, Bic-P_i_ and Bic-P_o_) would be lower in soils under meadow than heath vegetation, concomitant with higher Al and Fe concentrations and soil P sorption capacity. Our results partially support this, as overall Bic-P_o_ concentration was significantly lower, and sorption index and Al_ox_ + Fe_ox_ concentration were significantly higher, in meadow than in heath soils. Nevertheless, the concentration of Bic-P_i_ showed the opposite pattern and the concentration of Resin-P, the largest labile P pool, was not significantly different between vegetation types. Because higher Al and/or Fe has been shown to be positively correlated with P retention in organic soils [60], [76]–[78], we expected that it would also lead to lower concentrations of Resin-P, which has a high propensity for sorption. However, Al_ox_ and Fe_ox_ concentrations in our soils were much lower than those of other organic soils for which negative correlations between labile inorganic P and Al and/or Fe have been reported [60], [61], [76]. No correlations have been reported for other organic soils that have Al_ox_ and Fe_ox_ concentrations more within the range of what we measured here [6], [40]. Taken together, our results suggest that the Al and Fe concentrations in meadow soils are insufficient to exert a strong control on the concentration of labile inorganic P.

The higher Al_ox_ + Fe_ox_ concentrations and sorption index values in the meadow soils had little apparent effect on the concentration of Resin-P, but they may have resulted in higher sorption of organic P. This is suggested by the fact that concentrations of NaOH-P_o_ and Residual P (which in our soils is mostly organic) were 2.0– and 1.5– fold higher in meadow than in heath. Some organic P compounds have a high affinity for Al and Fe oxides [79]–[81], and soil organic P is often strongly positively related with Al and Fe concentration in organic soils, as seen in both this (Table 3) and other studies [40], [45], [61], [77], [82], [83]. Soil organic P is generally considered to be less prone to sorption than are labile inorganic P forms such as Resin-P [84], but in some humus soils organic P is correlated with Al_ox_ and Fe_ox_ concentration while labile inorganic P is not [40], which is consistent with our findings. A higher organic P sorption capacity in meadow soils could lower P availability indirectly by protecting organic P from microbial mineralization. This is supported by our results showing that Resin-P concentration expressed as a percentage of total soil P was approximately half in meadow than in heath soils (Table S2), and is consistent with findings of relatively higher P limitation in meadow than in heath vegetation [6], [13], [25].

### Conclusions

A 500 m decrease in elevation accompanied by a 2.5–3.0°C increase in temperature resulted in approximately 10–fold higher Resin-P concentration in subarctic soils under contrasting tundra vegetation types. However, the specific way in which Resin-P concentration changed with elevation (and thus temperature) differed with vegetation type. In meadow soils, the higher concentrations of oxalate-extractable Al and Fe, higher P sorption capacity, higher accumulation of organic P and proportionally lower concentrations of Resin-P than in heath soils is consistent with previous reports of higher relative P limitation in meadow vegetation. Our results suggest that predicted temperature increases of 3–5°C for the arctic in the next century could increase the concentrations of labile P in soils, but that the specific pattern of this increase is likely to depend on vegetation type. This is supported by previous work showing significant relationships between temperature and soil and vegetation properties known to influence P availability. Our results also emphasize the need for a better mechanistic understanding of P dynamics in arctic environments. Specifically, knowledge of organic P forms and of the processes that affect their mineralization is crucial for furthering our understanding of how temperature and vegetation affect P availability. New methods which apply 2-D ^1^H-^31^P NMR spectroscopy on soils [65] used in combination with enzyme assays and tracer experiments with ^32^P and ^33^P radioisotopes would be a natural follow-up of this study. This knowledge is important because future increases in soil P availability may affect key ecosystem processes such as primary production in these highly nutrient-limited tundra ecosystems.

## Supporting Information

Figure S1 **Temperature along the elevational gradient in 2009.** Daily mean temperature (°C) in July and August 2009 at 500 m, 700 m and 1000 m, along the elevational study gradient.
(TIF)

Figure S2 **Temperature along the elevational gradient in 2008.** Daily mean temperature (°C) from 28 June to 31 August 2008 measured at 400, 700 and 1000 m along the elevational study gradient.
(TIF)

Figure S3 **Concentration of soil phosphorus fractions divided by phosphorus sorption index along the elevational gradient.** Panels represent phosphorus (P) fractions extractable with: anion-exchange resins (Resin-P) and NaHCO_3_ (inorganic fraction – Bic-P_i_; and organic fraction – Bic-P_o_). Bars represent mean values (+1 SE) for four plots; for each P fraction and within each vegetation type, F and *p* values (with d.f.) are from a one-way ANOVA testing for the effect of elevation within each vegetation type, and bars topped with the same letter do not differ at *p* = 0.05 (Tukey's h.s.d.). Note the difference in y-axis scales.
(TIF)

Table S1 **Selected properties of humus soils in contrasting vegetation types along the elevational gradient.** Values represent the mean (±1 SE) of four plots, data from [13], [25].
(DOCX)

Table S2 **Concentrations of different phosphorus fractions across an elevational gradient.** Concentrations are expressed as proportions (%) of total soil phosphorus (P) in humus soils in contrasting vegetation types (heath and meadow). P_o_ =  organic P, P_i_ =  inorganic P. Values represent the means (±1 SE) of four replicate plots.
(DOCX)

Table S3 **Linear regressions between soil phosphorus fractions and air temperature.** Temperature is the August 2009 mean, and phosphorus (P) is measured on humus soils collected under each of two vegetation types along an elevational gradient.
(DOCX)
