# Supplementary figures and images for: Bioavailable Soil Phosphorus Decreases with Increasing Elevation in a Subarctic Tundra Landscape
Source: PLoS One. 2014 Mar 27;9(3):e92942. doi: 10.1371/journal.pone.0092942 (PMC3968050; doi:10.1371/journal.pone.0092942)

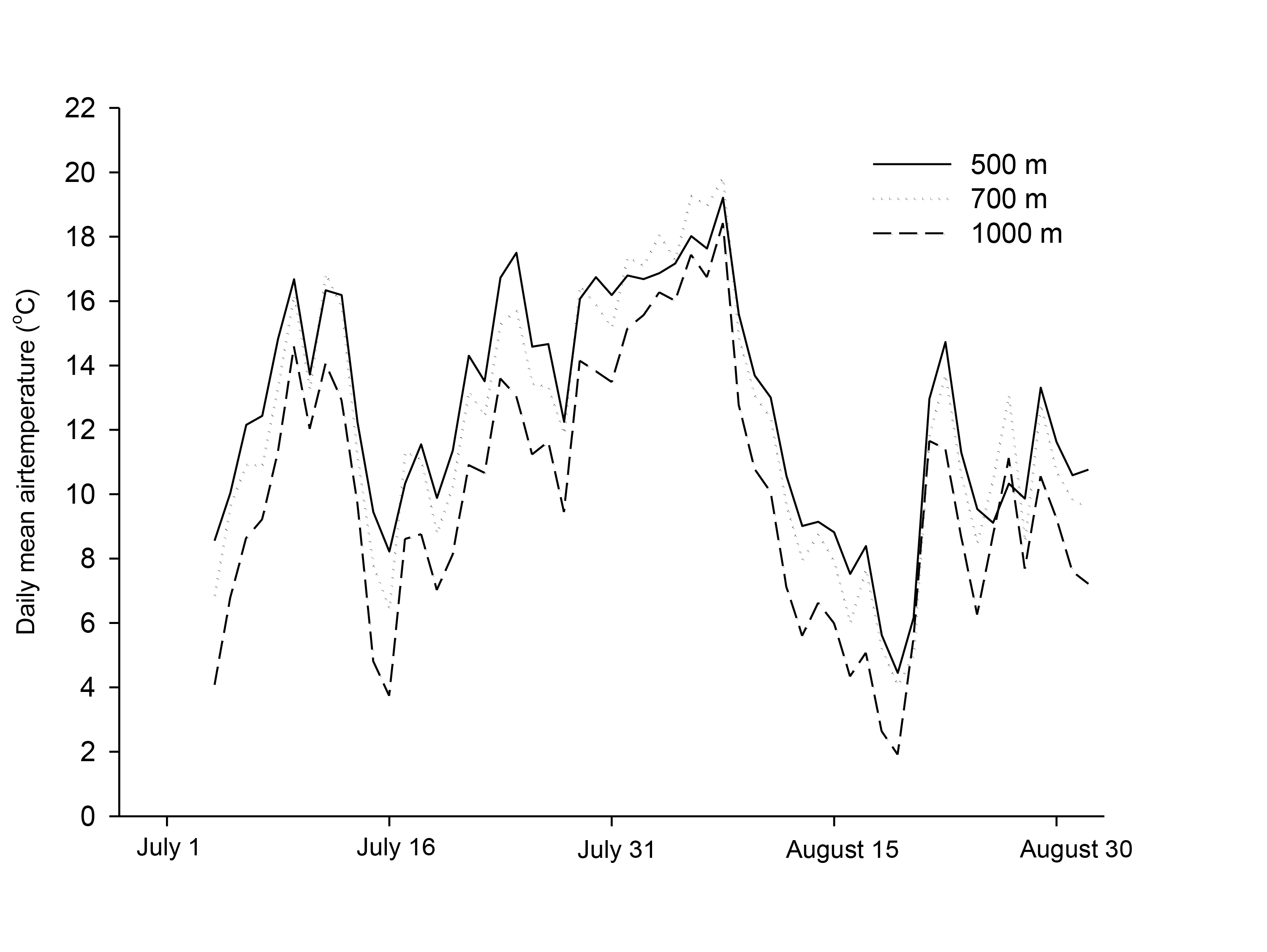

Supplement: Figure S1 — Temperature along the elevational gradient in 2009. Daily mean temperature (°C) in July and August 2009 at 500 m, 700 m and 1000 m, along the elevational study gradient. (TIF) [file pone.0092942.s001.tif]

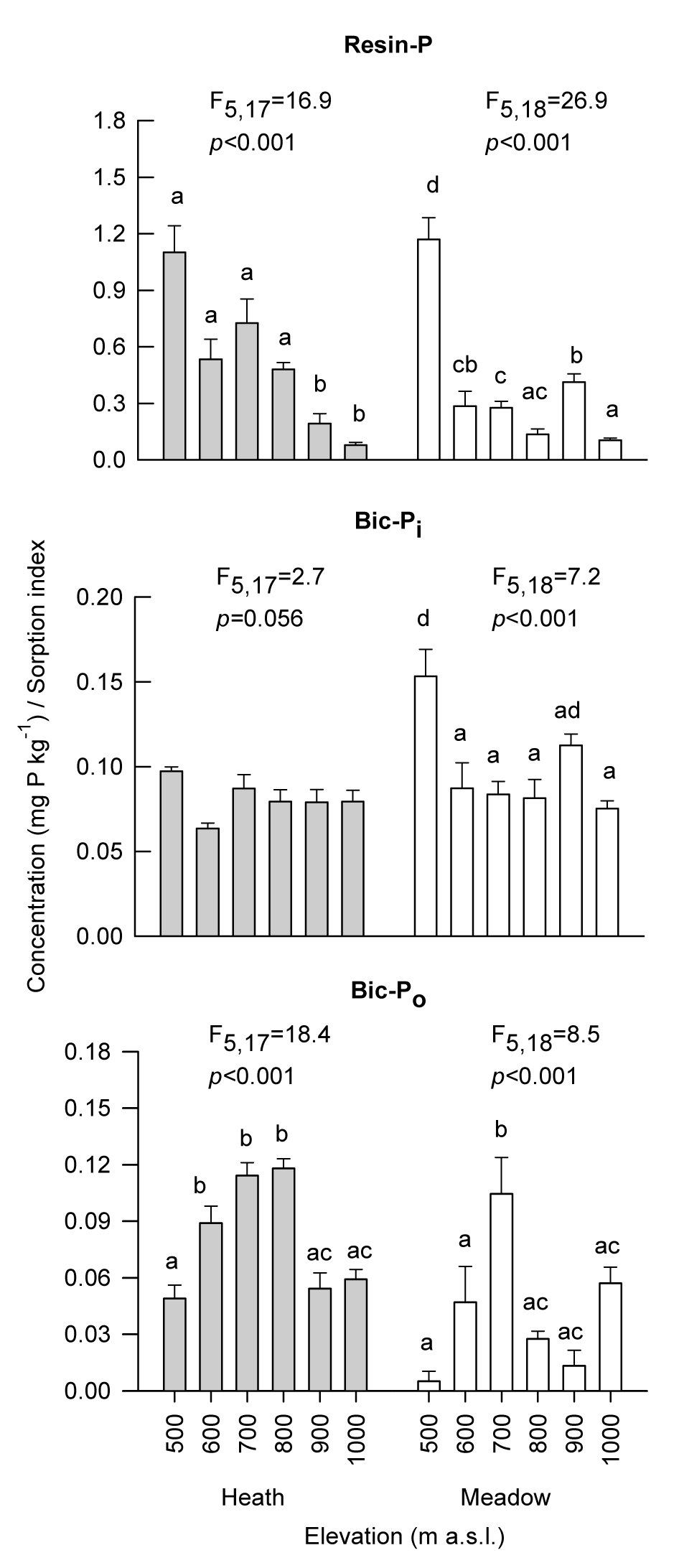

Supplement: Figure S3 — Concentration of soil phosphorus fractions divided by phosphorus sorption index along the elevational gradient. Panels represent phosphorus (P) fractions extractable with: anion-exchange resins (Resin-P) and NaHCO3 (inorganic fraction – Bic-Pi; and organic fraction – Bic-Po). Bars represent mean values (+1 SE) for four plots; for each P fraction and within each vegetation type, F and p values (with d.f.) are from a one-way ANOVA testing for the effect of elevation within each vegetation type, and bars topped with the same letter do not differ at p = 0.05 (Tukey's h.s.d.). Note the difference in y-axis scales. (TIF) [file pone.0092942.s003.tif]
