# Supplementary material for: Bioavailable Soil Phosphorus Decreases with Increasing Elevation in a Subarctic Tundra Landscape
Source: PLoS One. 2014 Mar 27;9(3):e92942. doi: 10.1371/journal.pone.0092942 (PMC3968050; doi:10.1371/journal.pone.0092942)
Supplement: Table S1 — Selected properties of humus soils in contrasting vegetation types along the elevational gradient. Values represent the mean (±1 SE) of four plots, data from [13], [25]. (DOCX) [file pone.0092942.s004.docx]

**Table S1. Selected properties of humus soils in contrasting vegetation types along the elevational gradient.** Values represent the mean (± 1 SE) of four plots, data from [13, 25].

| **Vegetation type** | **Elevation (m a.s.l.)** | **Litter N/P** | **Foliar N/P** | **pH** | **C/N** | **C/P** | **N/P** | **OM (%)** | **Ca (g kg^-1^)** |
| --- | --- | --- | --- | --- | --- | --- | --- | --- | --- |
| Heath | 500 | 8.6±02a | 8.8±0.3a | 4.5±0.1 | 32±1.4a | 494±92a | 16±3.1ab | 94±0.4 | 3.4±0.2bc |
|  | 600 | 8.9±0.7a | 8.0±0.6ab | 4.6±0.1 | 24±1.4b | 395±24a | 17±1.3ab | 75±3.8 | 3.2±0.9bc |
|  | 700 | 8.2±0.4a | 8.1±0.3a | 4.6±0.1 | 29±0.5a | 410±18a | 14±0.4b | 86±4.5 | 2.6±0.4c |
|  | 800 | 8.7±0.1a | 6.6±0.3b | 4.5±0.0 | 31±0.8a | 451±20a | 15±0.3ab | 91±1.1 | 1.9±0.1c |
|  | 900 | 11±0.6b | 12±0.3c | 5.3±0.2 | 23±1.4b | 391±34a | 17±0.4ab | 81±1.3 | 9.3±2.2a |
|  | 1000 | 13±0.5c | 13±0.8c | 5.0±0.1 | 24±1.3b | 448±39a | 19±1.2a | 87±1.2 | 5.2±0.6ab |
| Meadow | 500 | 11±1.3ab | 7.6±0.7a | 5.9±0.2 | 14±0.8a | 293±43a | 16±1.7ab | 80±1.4 | 13±4.6a |
|  | 600 | 9.7±0.9a | 9.4±0.8a | 5.3±0.2 | 16±0.4b | 216±15bc | 14±1.1bc | 56±4.4 | 5.7±1.2bc |
|  | 700 | 10±0.4ab | 9.0±0.3a | 5.3±0.1 | 15±0.5b | 173±9.0c | 12±0.4c | 60±3.5 | 3.9±0.5c |
|  | 800 | 12±0.3bc | 9.9±1.5a | 5.5±0.1 | 15±0.8b | 215±8.3bc | 14±1.0bc | 43±4.8 | 4.3±0.2ab |
|  | 900 | 15±0.4cd | 15±1.2b | 6.4±0.1 | 16±0.3b | 252±16ab | 16±1.0ab | 70±1.5 | 11±1.6a |
|  | 1000 | 15±0.4d | 22±1.7c | 5.5±0.1 | 16±0.4b | 279±18a | 17±0.7a | 60±1.1 | 5.5±0.4c |

Note: For each vegetation type within each column, values with the same letters are not significantly different at *p* = 0.05. All post-hoc tests were LSD tests with a Benjamini-Hochberg correction, except for litter N/P and foliar N/P which were Tukey’s h.s.d.
