# Supplementary material for: Bioavailable Soil Phosphorus Decreases with Increasing Elevation in a Subarctic Tundra Landscape
Source: PLoS One. 2014 Mar 27;9(3):e92942. doi: 10.1371/journal.pone.0092942 (PMC3968050; doi:10.1371/journal.pone.0092942)
Supplement: Table S2 — Concentrations of different phosphorus fractions across an elevational gradient. Concentrations are expressed as proportions (%) of total soil phosphorus (P) in humus soils in contrasting vegetation types (heath and meadow). Po = organic P, Pi = inorganic P. Values represent the means (±1 SE) of four replicate plots. (DOCX) [file pone.0092942.s005.docx]

**Table S2. Concentrations of different phosphorus fractions across an elevational gradient.** Concentrations are expressed as proportions (%) of total soil phosphorus (P) in humus soils in contrasting vegetation types (heath and meadow). P_o_ = organic P, P_i_ = inorganic P. Values represent mean values (± 1 SE) of four plots.

| **Vegetation type** | **Elevation (m a.s.l.)** | **Resin-P** | **Bicarbonate-extractable P** | | **Total labile P**^a^ | **NaOH-extractable P** | | **HCl-extractable P** | **Residual P** |
| --- | --- | --- | --- | --- | --- | --- | --- | --- | --- |
|  |  |  | **P_i_** | **P_o_** |  | **P_i_** | **P_o_** |  |  |
| Heath | 500 | 18±1.3 | 1.6±0.1 | 0.8±0.1 | 20±0.8 | 1.7±0.1 | 1.6±0.2 | 0.8±0.1 | 76±1.2 |
|  | 600 | 9.6±1.6 | 1.2±0.0 | 1.7±0.3 | 13±0.9 | 2.0±0.1 | 13±3.1 | 0.8±0.1 | 72±1.7 |
|  | 700 | 16±2.3 | 1.9±0.1 | 2.4±0.2 | 20±1.1 | 2.2±0.2 | 9.2±1.1 | 0.9±0.1 | 68±2.8 |
|  | 800 | 11±0.9 | 1.9±0.1 | 2.8±0.0 | 16±0.7 | 2.1±0.1 | 9.5±1.8 | 1.0±0.0 | 72±2.4 |
|  | 900 | 3.7±1.3 | 1.5±0.2 | 1.0±0.2 | 7±0.9 | 2.1±0.1 | 17±4.4 | 0.9±0.1 | 73±2.7 |
|  | 1000 | 1.5±0.3 | 1.5±0.3 | 1.2±0.2 | 4.3±0.6 | 2.0±0.1 | 22±2.2 | 0.8±0.0 | 71±2.0 |
| Meadow | 500 | 11±1.2 | 1.5±0.1 | 0.1±0.1 | 13±0.8 | 2.2±0.3 | 22±3.8 | 0.9±0.1 | 62±2.5 |
|  | 600 | 4.5±1.2 | 1.4±0.2 | 0.8±0.3 | 6.7±0.8 | 2.3±0.1 | 27±3.1 | 1.3±0.5 | 62±2.6 |
|  | 700 | 4.5±0.5 | 1.4±0.2 | 1.7±0.3 | 7.7±0.6 | 2.7±0.3 | 23±2.0 | 1.2±0.1 | 66±2.5 |
|  | 800 | 2.8±0.4 | 1.7±0.2 | 0.6±0.1 | 5.0±0.6 | 1.8±0.3 | 31±5.3 | 3.0±0.6 | 59±4.9 |
|  | 900 | 4.7±0.3 | 1.3±0.1 | 0.1±0.1 | 6.2±0.5 | 1.6±0.1 | 21±3.9 | 1.5±0.2 | 70±3.9 |
|  | 1000 | 1.7±0.3 | 1.2±0.1 | 0.9±0.1 | 3.7±0.4 | 2.6±0.2 | 30±3.5 | 1.2±0.1 | 63±3.4 |

^a^ Total labile P is calculated as the sum of Resin-P, Bicarbonate P_i_ and P_o._
