# Supplementary material for: Bioavailable Soil Phosphorus Decreases with Increasing Elevation in a Subarctic Tundra Landscape
Source: PLoS One. 2014 Mar 27;9(3):e92942. doi: 10.1371/journal.pone.0092942 (PMC3968050; doi:10.1371/journal.pone.0092942)
Supplement: Table S3 — Linear regressions between soil phosphorus fractions and air temperature. Temperature is the August 2009 mean, and phosphorus (P) is measured on humus soils collected under each of two vegetation types along an elevational gradient. (DOCX) [file pone.0092942.s006.docx]

**Table S3. Linear regressions between soil phosphorus fractions and air temperature.** Temperature is the August 2009 mean, and phosphorus (P) is measured on humus soils collected under each of two vegetation types along an elevational gradient.

| **P fraction** |  | **Heath** |  |  |  | **Meadow** |  |
| --- | --- | --- | --- | --- | --- | --- | --- |
|  | **R^2^** | ***p*** | **Direction** |  | **R^2^** | ***p*** | **Direction** |
| Resin P | **0.682** | **<0.001** | **Positive** |  | **0.255** | **0.007** | **Positive** |
| Bic-extractable P_i_ | 0.027 | 0.797 | N/A |  | 0.014 | 0.418 | N/A |
| Bic-extractable P_o_ | 0.001 | 0.688 | N/A |  | 0.044 | 0.843 | N/A |
| Total labile P ^a^ | **0.648** | **<0.001** | **Positive** |  | **0.277** | **0.005** | **Positive** |
| NaOH-extractable P_i_ | 0.000 | 0.323 | N/A |  | 0.045 | 0.940 | N/A |
| NaOH-extractable P_o_ | **0.345** | **0.002** | **Negative** |  | 0.011 | 0.273 | N/A |
| HCl-extractable P | 0.045 | 0.918 | N/A |  | 0.005 | 0.301 | N/A |
| Residual P | 0.034 | 0.633 | N/A |  | 0.000 | 0.330 | N/A |
| Total P ^b^ | 0.032 | 0.592 | N/A |  | 0.018 | 0.448 | N/A |

Degrees of freedom for all variables for both vegetation types are 1,22.

^a^ Total labile P is calculated as the sum of Resin-P, Bicarbonate P_i_ and P_o._

^b^ Total P is calculated as the sum of all sequentially extracted P fractions.
